# Flavonoid-Rich Extract of *Paeonia lactiflora* Petals Alleviate d-Galactose-Induced Oxidative Stress and Restore Gut Microbiota in ICR Mice

**DOI:** 10.3390/antiox10121889

**Published:** 2021-11-26

**Authors:** Lei Liu, Yingdan Yuan, Jun Tao

**Affiliations:** 1 College of Animal Science and Technology, Yangzhou University, Yangzhou 225009, China; dx120170096@yzu.edu.cn; 2 College of Horticulture, Xinyang Agriculture and Forestry University, Xinyang 464000, China; 3 College of Horticulture and Plant Protection, Yangzhou University, Yangzhou 225009, China; yyd@yzu.edu.cn; 4 Joint International Research Laboratory of Agriculture and Agri-Product Safety, The Ministry of Education of China, Yangzhou University, Yangzhou 225009, China

**Keywords:** *Paeonia lactiflora*, flavonoids, oxidative stress, gut microbiota, d-galactose

## Abstract

This study was aimed to investigate the antioxidant effect of *Paeonia lactiflora* Pall. petal flavonoids extract (PPF) on d-galactose (d-gal)-induced ICR mice. In this study, sixty male ICR mice were randomly divided into six groups during an 8 weeks experimental period, including normal control (NC) group, d-gal group, epigallocatechin gallate (EGCG) group, low, medium, and high dose PPF groups (10, 20 and 40 mg/kg/day). The results showed that intragastric administration with PPF significantly reverses the atrophy of the visceral organs of oxidative damage mice in a dose-dependent relationship. PPF indicated the antioxidant capacity to decrease the malondialdehyde (MDA) level and improve the activity of superoxide dismutase (SOD), catalase (CAT) as well as glutathione peroxidase (GSH-Px). In addition, PPF treatment reversed gut microbiota dysbiosis by increasing the relative abundance of *Lactobacillaceae*. Spearman correlation analysis showed that the body’s oxidative stress markers were directly related to changes in gut microbiota. These findings reveal firstly that PPF could alleviate d-Gal-induced oxidative stress and modulate gut microbiota balance.

## 1. Introduction

The potential harm of oxidative stress to human beings and animals has attracted extensive attention. Oxidative stress is a gradual process from the time of birth [1], and the balance of the oxidation and antioxidants is broken with the gradual increase in reactive oxygen species (ROS) in the organism. Excessive oxidative stress might contribute to lipid peroxidation, DNA damage, inflammation responses, and ultimately lead to physiological malfunctions and diseases, such as diabetes, heart disease, cancer, and Parkinson’s and Alzheimer’s diseases [2,3]. Hence, maintaining the normal oxidation-reduction balance of the organism is essential to the overall health of human beings. According to the reported antioxidant mechanism, living organisms remove excessive oxygen free radicals by scavenging ROS precursors, chelating with metal ions involved in the formation of ROS, and increasing endogenous antioxidant defenses in the body [4]. Given the important impact of oxidative stress on living organisms, adding various antioxidant supplements into the daily diet is essential to antagonize oxidative stress and maintain normal body organs.

There are a vast number of microbes in the intestines of humans and animals, and they depend on and restrict each other with the host. After long-term evolution, they form a specific intestinal microecosystem with the host intestine, which participates in the host’s metabolism, growth, and immunity [5]. The gut microbiota is an essential link between diet and host physiology. On the one hand, the host obtains certain nutrients through intestinal microbial metabolism. On the other hand, the structure and function of the gut microbiota are critically affected by diet [6]. Increasing evidence suggested that these changes in the level of oxidative stress affected the microbial environment of the intestine and microbiota structure, which eventually caused inflammation and metabolic syndrome in living organisms [7].

*Paeonia lactiflora* Pall. belong to the Paeoniaceae family—a herbaceous perennial flowering plant with fleshy root and annual stem [8]. The skinless *P. lactiflora* root is called *Radix Paeoniae Alba*, a well-known traditional Chinese medicine for centuries. In addition to medicinal value, *P. lactiflora* has significant ornamental value, cultivated widely all over the world. However, many petals remain underutilized systematically after the flowering period due to underestimating its potential use, causing substantial waste of resources. Previous studies indicated that the extract from the petals of *P. lactiflora* was high in flavonoids, and it had demonstrated that the extract exerts antioxidant activity in vitro. However, litter is known about the underlying mechanisms by which flavonoids from *P. lactiflora* petals alleviate oxidative stress in vivo, and particularly about their effect on the gut microbiota. As a result of our previous research in model organisms, we hypothesized that *P. lactiflora* petal flavonoids may have an effect on the antioxidant activity and gut microbiota of animals, and further investigation is required.

The d-Galactose-induced subacute aging mouse model is widely used in organ aging research and drug testing. d-gal can be converted to galactitol after prolonged injection of high-dose. Because galactitol cannot be decomposed, and accumulates in the cells, leading to enhanced ROS eventually [4,9,10]. The present study evaluated the antioxidant capacity of PPF in vivo, investigated the underlying mechanism, and explored the correlation between the gut microbiota and antioxidant status in the d-gal-induced oxidative stress mouse model. Our results may provide a novel scientific basis for the development and application of *P. lactiflora* petal.

## 2. Materials and Methods

### 2.1. Paeonia lactiflora Flavonoids Extraction and Characterization

*P. lactiflora* ‘Hong Yan Zheng Hui’ was artificially cultivated in the herbaceous peony germplasm nursery at Yangzhou University, Jiangsu Province, China (Latitude, 32°39′ N; Longitude, 119°42′ E). The flowers of herbaceous peony were collected in early May 2020. After the stamens and ovary were removed, and the petals were dried in an oven at 45 °C, ground into a powder with an electric mill, and passed through a fine sieve of 80 mesh. 

The extraction and purification of *P. lactiflora* petal flavonoids were based on the method of Zhang et al. [11], with slight modifications. The *P. lactiflora* petals were extracted with 95% aqueous ethanol solution (1:20), ultrasonically extracted at 25 °C for 30 min, the extraction was repeated three times, and the three filtrates were combined. Ethanol aqueous solution was removed with a rotary evaporator. The crude extracts were diluted with ultrapure water and filtered with a 0.8 μm filter membrane. The crude extract was loaded into a Sep-Pak^®^ C18 cartridge (12 cc/2 g, Waters) to remove polysaccharides and then eluted with methanol (Figure S1). The eluent was evaporated under reduced pressure to obtain *P. lactiflora* petal flavonoids extract (PPF). The chemical components and content of PPF are listed in Table S1. The chemical structures of some main compounds in PPF are listed in Figure S2.

### 2.2. Reagents 

d-galactose and epigallocatechin gallate (EGCG) was obtained from Sigma-Aldrich (St. Louis, MO, USA). Physiological saline was purchased from Anhui BBCA Pharmaceutical Co., Ltd. (Hefei, Anhui, China). The superoxide dismutase (SOD), catalase (CAT), glutathione peroxidase (GSH-Px), malondialdehyde (MDA) assay kits, and BCA protein assay kit were obtained from Nanjing Jiancheng Institute of Biotechnology (Nanjing, Jiangsu, China). Formalin neutral fixative (*v*/*v*, 10%) was purchased from Nanchang Yulu Experimental Equipment Co., Ltd. (Nanchang, Jiangxi, China). The water used in the study was obtained from a Milli-Q Plus ultrapure purification system (Bedford, MA, USA). All other reagents were standard analytical grade.

### 2.3. Animal Experimental Design and Ethics Statement

A total of 60 male six-week-old ICR mice were purchased from the Comparative Medicine Center of Yangzhou University. The mice were placed in a specific pathogen-free (SPF) room with a temperature of 20–22 °C, 60–75% relative humidity, alternative 12 h light/dark cycles, and given free access to food and water. The mice were fed a granular irradiated diet purchased from Jiangsu Xietong Pharmaceutical Bio-Engineering Co., Ltd. (Nanjing, Jiangsu, China). The diet contained protein (200 g/kg), fat (40 g/kg), crude fiber (50 g/kg), crude ash (80 g/kg), calcium (18 g/kg), phosphorus (12 g/kg), lysine (13.2 g/kg), and methionine and cystine (7.8 g/kg). After a one-week acclimatization period, the mice were randomly divided into six groups (*n* = 10 per group and 5 mice per cage in the same group): normal control (NC) group with saline by intragastrical gavage (0.1 mL/10 g), d-gal induced group with the same volume of saline, positive control group (EGCG) with 10 mg/kg b.w./day EGCG (dissolved in saline) by intragastrical gavage (0.1 mL/10 g). Low-dose group (PPF-L), medium-dose group (PPF-M), high dose group (PPF-H) with 10 mg/kg b.w./day, 20 mg/kg b.w./day, 40 mg/kg b.w./day PPF (dissolved in saline) by intragastrical gavage (0.1 mL/10 g), respectively. Apart from the NC group, all the mice were intraperitoneally injected with d-gal (100 mg/kg b.w./day). The mice were weighed once a week, observed the general condition of the animals, adjusted the dosage according to the last bodyweight, and continued to administer for eight weeks.

At the end of the experiment, the mice were fasted for 12 h before being killed (Just feed water), and the blood was collected by eyeball removal. The blood was allowed to stand at 4 °C for 8 h to coagulate and then centrifuged at 4 °C and 3000 r/min for 10 min. Serum samples were collected for the determination of biochemical indicators. The mice were sacrificed by cervical dislocation. Liver tissue was removed, washed with cold normal saline, and sub-packaged into 2 mL cryopreservation tubes. The cecum content of 60 mice was collected in sterile tubes, respectively, used to analyze gut microbiota. All the experimental samples were stored at −80 °C before corresponding analysis. During the experiment, the animal management strictly abides by the regulations of the experimental animal ethics committee of Yangzhou University on the care and use of experimental animals.

### 2.4. Determination of Liver and Serum Antioxidant Indexes

Liver tissues were added with pre-cooled saline at a ratio of 1:9 (m/V), homogenized in an ice bath to prepare 10% (*w*/*v*) homogenate. The homogenate was centrifuged at 4000 rpm for 10 min at 4 °C, and the supernatant was used to determine tissue biochemical indicators. SOD, CAT, GSH-Px activity, and MDA content of liver and serum were determined according to the operation steps of the kit. The BCA kit was used to determine the protein content of the sample at 562 nm.

### 2.5. Histological Observations

The liver tissues (0.5 cm^2^) were extracted and fixed in 10% formalin solution at room temperature for 48 h. The tissues were dehydrated by gradient ethanol, transparent with xylene, and then embedded in paraffin. The samples were cut into five μm thick sections and stained with hematoxylin and eosin (H&E). Histopathological changes in the liver were observed using an optical microscope (Nikon, Tokyo, Japan).

### 2.6. Analysis of Organ Coefficient

All mice were sacrificed by cervical dislocation. The thymus, brain, heart, liver, spleen, and kidney were thoroughly separated and weighed to calculate the organ coefficient [12]. Organ coefficient = organ weight (mg)/body weight (g) × 1000.

### 2.7. Gut Microbiota Analysis

Genomic DNA was extracted using HiPure Stool DNA Kit (Guangzhou Magen Biotechnology Co., Ltd., Guangzhou, China). The extracted DNA concentration and purity were detected by NanoDrop 2000. The integrity of DNA was examined by 1% agarose gel electrophoresis. The V3–V4 region (~466 bp) of bacterial 16S rDNA was amplified using barcode-specific primers. The primers were 341F (5′-CCTACGGGNGGCWGCAG-3′) and 806R (5′-GGACTACHVGGGTATCTAAT-3′). The amplicons were purified using AMPure XP Beads and quantified using the ABI Step One Plus Real-Time PCR System (Life Technologies, CA, USA). Paired-end amplicon sequencing was performed using the Illumina NovaSeq 6000 sequencing platform (Gene Denovo Biotechnology, Guangzhou, China). The GM sequence data had been deposited at NCBI Sequence Read Archive Database under accession number SRP345581. After reads splicing and filtering, the obtained data were subjected to species annotation and abundance analysis.

### 2.8. Statistical Analysis

SPSS 20.0 software was used for statistical analysis (IBM, New York, NY, USA). The differences between multiple groups were analyzed using a one-way ANOVA followed by Duncan’s multiple comparisons test. The experimental data were expressed as the mean ± standard deviation, *p*-values less than 0.05 were regarded as statistically significant. Spearman correlation coefficient (r) was evaluated to determine the correlation between antioxidant biomarkers and gut microbes. GraphPad Prism 8.0 software was used for drawing.

## 3. Results

### 3.1. Effect of PPF on the Organ Index of Mice

In biomedical research, the organ index can reflect the changes in animal organs or tissue structure, which is an important indicator to measure the functional status of animals. Changes in the visceral tissue index of mice before and after the modeling and administration are shown in Table 1. Compared with the NC group, the tissue index of the heart, brain, liver, kidney, thymus, and spleen of the d-gal group were all significantly reduced (*p* < 0.05), indicating that the visceral organs of mice atrophied in varying degrees caused by d-gal. The EGCG, PPF-M, and PPF-H groups could significantly reverse the atrophy of the visceral organs in a dose-dependent relationship. PPF had a better antagonistic effect on mice’s visceral organ atrophy and immune enhancement caused by oxidative damage.

### 3.2. Histopathological Observation of Liver Tissue

**Figure 1:**
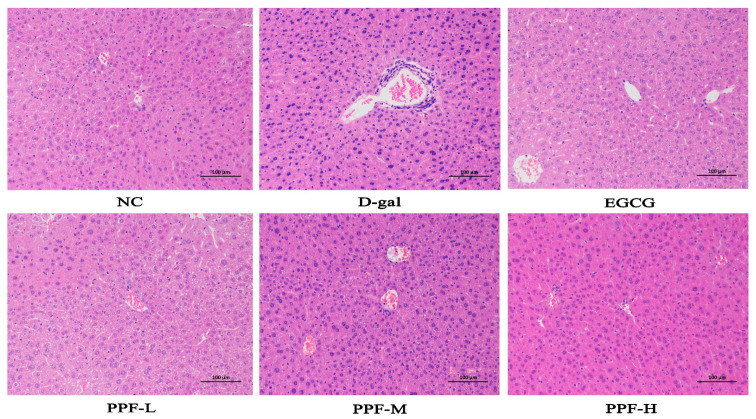
Histopathological observation of liver in the mice injured by d-galactose. NC, normal control group; d-gal, d-galactose model group; EGCG, 10 mg/kg b.w./day epigallocatechin gallate; PPF-L, 10 mg/kg b.w./day *P. lactiflora* flavonoids extract; PPF-M, 20 mg/kg b.w./day *P. lactiflora* flavonoids extract; PPF-H, 40 mg/kg b.w./day *P. lactiflora* flavonoids extract.

The liver plays a vital role in protecting the organism from potentially harmful chemical damage [13,14]. Some changes in liver morphology were discovered in the d-gal-induced oxidative stress model of mice. The results of liver hematoxylin-eosin staining in each group were shown in Figure 1. The hepatocytes of the NC group were arranged radially along the central vein, and the morphology was regular and orderly. The hepatocytes were the same size and there was no sign of inflammatory cell aggregation. Mild degeneration and inflammatory infiltration of cells were discovered around the portal area in the d-gal group. Moreover, with the increase in PPF dosage, the improvement effect became better. Histopathological evaluation results showed that PPF had a protective effect on liver oxidative stress damage caused by d-galactose.

### 3.3. Analysis of Antioxidant Activity In Vivo

**Figure 2:**
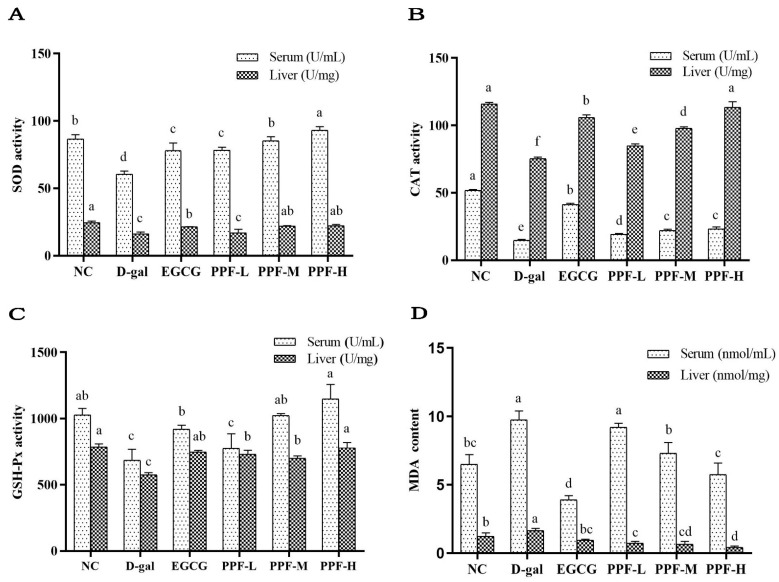
Biochemical indicators of serum and liver of experimental mice. (**A**) SOD activity; (**B**) CAT activity; (**C**) GSH-Px activity; (**D**) MDA content. Mean values with different letters in the bar graph of the same sample were significantly different (*p* < 0.05) according to Duncan’s multiple range test.

SOD activity of serum and liver in the d-gal group was significantly lower than that in the NC group, indicating that the d-gal-induced oxidative stress model was constructed successfully. SOD activity of serum in the PPF-L, PPF-M, and PPF-H groups and the EGCG group significantly increased in a concentration-dependent manner compared with the d-gal group (*p* < 0.05). There was no significant difference in the SOD activity of the liver between the PPF-L group and the d-gal group. In contrast, SOD activity significantly increased in the PPF-M group and PPF-H group (Figure 2A).

As shown in Figure 2B, the CAT activity of the serum and liver in the model group was significantly lower than that of the NC group (*p* < 0.05). The low, medium, and high dose groups of PFE can significantly increase CAT activity of serum and live. The GSH-Px activity in serum and liver of the d-gal group was significantly lower than that in the NC group and the EGCG group (Figure 2C). The PPF-L, PPF-M, PPF-H groups could significantly increase the GSH-Px activity of serum and liver compared with the d-gal group (*p* < 0.05), which showed a dose-dependent type in the serum sample. 

MDA content in serum and liver of mice in the d-gal group increased significantly compared with the NC group (Figure 2D), after the mice were gavaged different doses of PPF (10~40 mg/kg) significantly reduced the level of MDA (*p* < 0.05).

### 3.4. PPF Modulated the Structure of Gut Microbiota

**Figure 3:**
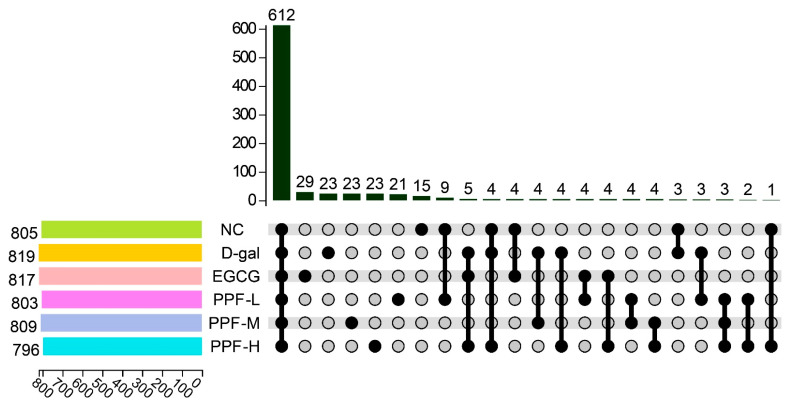
OTU Venn diagram in different treatment.

16S rRNA gene sequencing was used to analyze the structure of gut microbiota in the cecum of mice. Samples in different groups had specific characteristics and commonalities. This research group compared the common and unique OTU situations of the six treatment groups, as shown in Figure 3, the six treatment groups had a total of 612 common OTUs, the NC group had 15 unique OTUs, the d-gal group had 23 unique OTUs, and the EGCG group had unique 29 OTUs. The doses of PPF-L, PPF-M, and PPF-H had 21, 23, and 23 unique OTUs, respectively.

**Figure 4:**
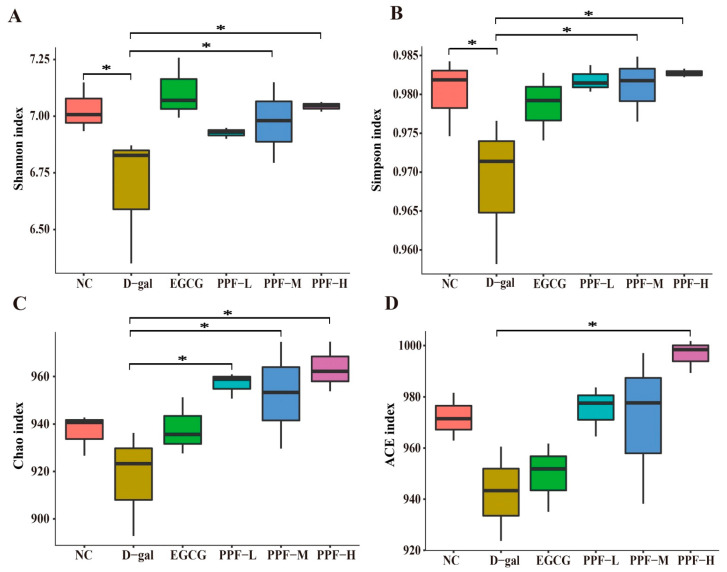
Analysis of alpha diversity at the level of the operational taxonomic unit. An asterisk (*) indicates a significant difference from the d-gal group (*p* < 0.05).

The alpha diversity of gut microbiota is shown in Figure 4. The Shannon index (6.68 ± 0.29), Simpson index (0.968 ± 0.009), and Chao index (917.43 ± 22.31) in the d-gal group were significantly lower than those in the NC group (*p* < 0.05), indicating that the richness and uniformity of gut microbiota decreased significantly after mice experienced oxidative stress. The alpha diversity index of the different dose PPF groups was higher than that of the d-gal group, and especially the PPF-H group had the highest alpha diversity. Interestingly, the ACE index of the PPF-H group was significantly higher than that of the EGCG group. Based on the above data analysis, it could be seen that d-gal-induced oxidative stress mice could significantly increase the diversity of gut microbiota after being fed PPF.

**Figure 5:**
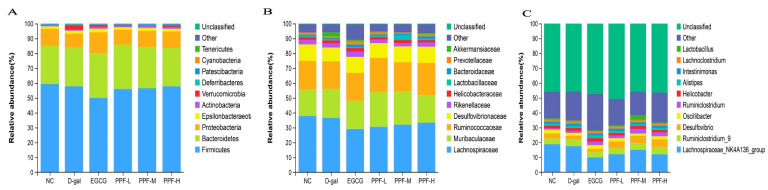
Composition of gut microbiota at different taxa levels. (**A**) Phylum-level; (**B**) Family- level; (**C**) Genus -level.

The assigned sequence reads were used to evaluate species composition at different taxonomic levels. The species with the average abundance of top 10 were displayed in all samples, and the remaining species were unified into the other category, and tags that could not be annotated to this level were classified into the unclassified category. At the phylum level (Figure 5A), *Firmicutes*, *Bacteroidetes*, and *Proteobacteria* accounted for about 95% of the total gut microbiota. Compared with the NC group, the relative abundances of *Verrucomicrobia* and *Tenericutes* in the d-gal group were increased. The relative abundances of *Verrucomicrobia* and *Tenericutes* returned to the level of the NC group after the mice were fed high doses of PPF. At the family level (Figure 5B), compared to the NC group, the relative abundance of *Ruminococcaceae*, *Desulfovibrionaceae*, *Lactobacillaceae*, and *Prevotellaceae* decreased in the d-gal group. In contrast, the relative abundance of their microbiota increased in the EGCG group and the PPF-H group. The relative abundances of *Helicobacteraceae* and *Akkermansiaceae* increased in the d-gal group, while the relative abundances of the two microbiota returned to NC group level after high-dose PPF feeding. At the genus level (Figure 5C), d-Gal intraperitoneal injection reduced the proportion of *Desulfovibrio*, *Oscillibacter*, *Lactobacillus*. It increased the proportion of *Helicobacter*, *Intestinimonas* in the d-gal group compared with the NC group. After intervention with high-dose PPF, the relative abundances of the above five genera returned to the NC group level. Compared to the d-gal group, the relative abundance of *Lachnospiraceae*_NK4A136_group decreased, and the relative abundance of *Ruminiclostridium* increased in the PPF-H group.

**Figure 6:**
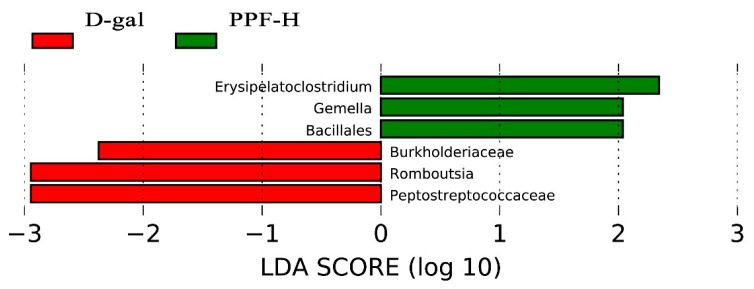
The taxa with LDA score from LEfSe analysis.

LEFse (linear discriminant analysis effect size) was used to analyze the different species between the d-gal and the PPF-H groups. Figure 6 shows microorganisms with significant differences in abundance in two groups under the condition that the LDA score was more significant than the set value (the default setting was 2), that is, the abundance in the group was significantly higher than the biomarker of the other groups. The length of the histogram represented the impact of different species. There were *Burkholderiaceae*, *Romboutsia*, and *Peptostreptococcaceae* in the d-gal group. Unlike the d-gal group, the PPF-H group mainly enriches *Erysipelatoclostridium*, *Gemella*, and *Bacillales*.

### 3.5. Analysis of Correlation between Changes in Gut Microbiota and Antioxidant Indexes

**Figure 7:**
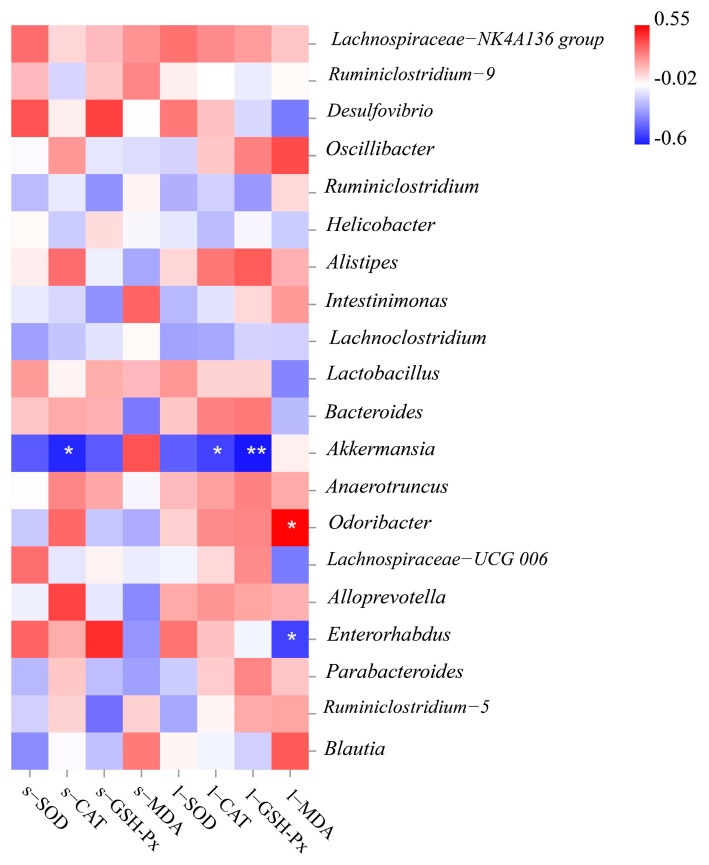
Heatmap of the correlation coefficient between the antioxidant index and bacterial taxa at the genus level.

We performed correlation analysis between the genus level of gut microbiota and oxidative stress biomarkers in six treatment groups, to evaluate the potential link between significantly altered taxa in the gut microbiota and oxidative stress-related parameters (Figure 7). Among the top 20 bacterial genera listed, three genera were significantly related to oxidative stress biomarkers in serum and liver. *Akkermansia* was negatively correlated with CAT activity in serum or liver, GSH-Px activity in the liver. *Enterorhabdus* was significantly negatively correlated with MDA content, and only *Odoribacter* was significantly positively correlated with MDA content in the liver.

## 4. Discussion

Clinical research shows that oxidative stress is related to various human diseases, such as neurodegenerative diseases, cardiovascular diseases, allergies, immune system dysfunction, diabetes, aging, cancer [15]. Various synthetic antioxidants have been questioned due to their safety and long-term effects [16]. Natural antioxidants are safe, non-toxic, and highly effective, so they are favored by consumers. Over the past decade, flavonoids in plants have attracted wide attention for their antioxidant activity [17]. Supplementation with a certain amount of natural antioxidants to the diet is relatively simple and effective for controlling oxidative stress [18]. The present study was the first work on the antioxidant activity of PPF in vivo by using the d-gal-induced oxidative stress model. EGCG, the most effective antioxidant polyphenol in green tea [19], was used as a positive control in this study.

Changes in organ index reflect the state of the body after oxidative stress. After continuous d-galactose administration, the brain and organs of mice shrank significantly, indicating that organs were damaged by oxidative stress to a certain extent, which might be related to the deterioration of multifactorial function [20]. Atrophy of the thymus and spleen suggests that the animal’s immune function could be reduced [21]. PPF alleviated the decrease in organ index caused by oxidative stress and also improved the immunity of mice. 

Excessive reactive oxygen free radicals are harmful to the organism. The function of the endogenous antioxidant system is to maintain the balance of the redox state and scavenge excessive ROS [22]. The endogenous antioxidant system includes antioxidant enzymes, SOD, GSH-Px, CAT, and antioxidants such as vitamin E and GSH [23,24]. These antioxidant enzymes activity can comprehensively reflect the antioxidant capacity of the organism [12]. SODs are universal enzymes for organisms living in oxygen, catalyzing the superoxide into oxygen and hydrogen peroxide. SODs are the first line of defense against oxidative stress [25]. CAT can promote the decomposition of H_2_O_2_ into molecular oxygen and water [26], whereas GSH-Px catalyzes the reduction in H_2_O_2_ or organic hydroperoxides to water or alcohols, respectively [27]. Malondialdehyde is the by-product of lipid peroxidation, and its concentration is widely used as a biomarker of the lipid peroxidation level [28]. The finding revealed that PPF could enhance SOD, GSH-Px, CAT activity, and reduce MDA content in serum and liver to resist ROS damage in this study, which is in agreement with the antioxidant function of flavonoids in other studies. The flavonoids extracted from indocalamus leaves and *Abelmoschus manihot* (L.) Medic flowers elevated the activity of antioxidant enzymes, decreased MDA production [29,30].

In this study, regulatory effects on the gut microbiota by PPF were discovered. The d-gal group resulted in a decrease in the diversity of gut microbiota. In the meantime, PPF supplementation increased the richness and diversity of gut microbiota in d-gal-induced oxidative stress mice, which might be related to the reproduction of some gut microorganisms promoted by PPF. In the analysis of the composition of gut microbiota, the abundance of *Lactobacillus* was increased by PPF. *Lactobacillus* spp. is a probiotic bacterium that presents antioxidant activity, scavenges superoxide anions and hydrogen peroxide, and improves total antioxidant stress in healthy subjects [31,32]. Thus, it is speculated that PPF may alleviate the oxidative damage induced by d-gal by increasing the expression abundance of *Lactobacillus* in the caecum of mice. Intraperitoneal injection of d-gal affected the changes in antioxidant indexes in serum and liver of mice and led to the imbalance of gut microbiota. Furthermore, in this study, correlation analysis showed that the body’s oxidative stress markers were directly related to changes in gut microbiota. This indicated that the regulation of PPF on the gut microbiota of d-gal-induced oxidative stress mice was linked to oxidative stress, however, a specific mechanism needs to be further studied. However, the lack of research on the bioavailability and drug metabolism of PPF in this study. It is necessary to focus on this aspect of research in the future, which will help to understand the antioxidant mechanism of PPF in vivo comprehensively.

## 5. Conclusions

The study revealed that *Paeonia lactiflora* petal flavonoids can alleviate oxidative stress by increasing the activity of SOD, CAT, and GSH-Px and decreasing the level of MDA in mice. The administration of PPF with potential antioxidant effects could effectively alleviate the organ atrophy and liver inflammatory cell infiltration induced by d-galactose. In addition, high-dose PPF treatment increased the abundance of *Lactobacillaceae* in d-gal-induced oxidative stress mice and maintained the balance of the entire gut microbiota. Our findings demonstrated PPF was related to significant alteration of the gut microbiota, which has the potential to be developed into a new type of antioxidant food.

## Figures and Tables

**Table 1:** Effects of different treatments on the visceral organ index of experimental mice. Values are presented as the mean ± standard deviation. Different letters indicated significant differences (*p* < 0.05) according to Duncan’s multiple range test. NC, normal control group; d-gal, d-galactose model group; EGCG, 10 mg/kg b.w./day epigallocatechin gallate; PPF-L, 10 mg/kg b.w./day *P. lactiflora* flavonoids extract; PPF-M, 20 mg/kg b.w./day *P. lactiflora* flavonoids extract; PPF-H, 40 mg/kg b.w./day *P. lactiflora* flavonoids extract.

Group	Index (mg/g·b.w.)					
	Heart	Brain	Liver	Kidney	Thymus	Spleen
NC	6.51 ± 0.34 ^a^	14.12 ± 0.40 ^a^	44.01 ± 1.66 ^a^	16.52 ± 1.46 ^a^	1.07 ± 0.14 ^a^	2.71 ± 0.14 ^a^
d-gal	4.14 ± 0.07 ^c^	10.31 ± 0.98 ^c^	31.51 ± 1.78 ^d^	10.77 ± 1.35 ^c^	0.25 ± 0.05 ^d^	1.26 ± 0.09 ^d^
EGCG	6.45 ± 1.01 ^a^	13.14 ± 0.18 ^ab^	39.43 ± 0.78 ^b^	14.49 ± 1.47 ^b^	0.59 ± 0.04 ^b^	2.29 ± 0.13 ^b^
PFE-L	5.01 ± 0.12 ^b^	10.60 ± 1.52 ^c^	34.54 ± 0.32 ^c^	10.93 ± 0.29 ^c^	0.42 ± 0.04 ^c^	1.68 ± 0.15 ^c^
PFE-M	5.29 ± 0.16 ^b^	12.41 ± 0.29 ^b^	39.05 ± 1.36 ^b^	12.97 ± 0.57 ^b^	0.68 ± 0.05 ^b^	1.81 ± 0.14 ^c^
PFE-H	6.07 ± 0.33 ^ab^	13.21 ± 0.69 ^ab^	40.31 ± 1.60 ^b^	14.08 ± 0.82 ^b^	0.69 ± 0.15 ^b^	2.18 ± 0.48 ^bc^

## Data Availability

The data is contained within the article.

## Supplementary Materials

The following are available online at https://www.mdpi.com/article/10.3390/antiox10121889/s1. Figure S1: Diagram for the obtainment of purified *Paeonia lactiflora* petal flavonoids; Figure S2: Chemical structures of some main compounds in *Paeonia lactiflora* petal flavonoids. Table S1: Flavonoid compounds components in *Paeonia lactiflora* petal extract (PPF).
