# Supplementary material for: Flavonoid-Rich Extract of Paeonia lactiflora Petals Alleviate d-Galactose-Induced Oxidative Stress and Restore Gut Microbiota in ICR Mice
Source: Antioxidants (Basel). 2021 Nov 26;10(12):1889. doi: 10.3390/antiox10121889 (PMC8698645; doi:10.3390/antiox10121889)
Supplement: Supplementary file 1 [file antioxidants-10-01889-s001.zip › antioxidants-1465616-supplementary.pdf]

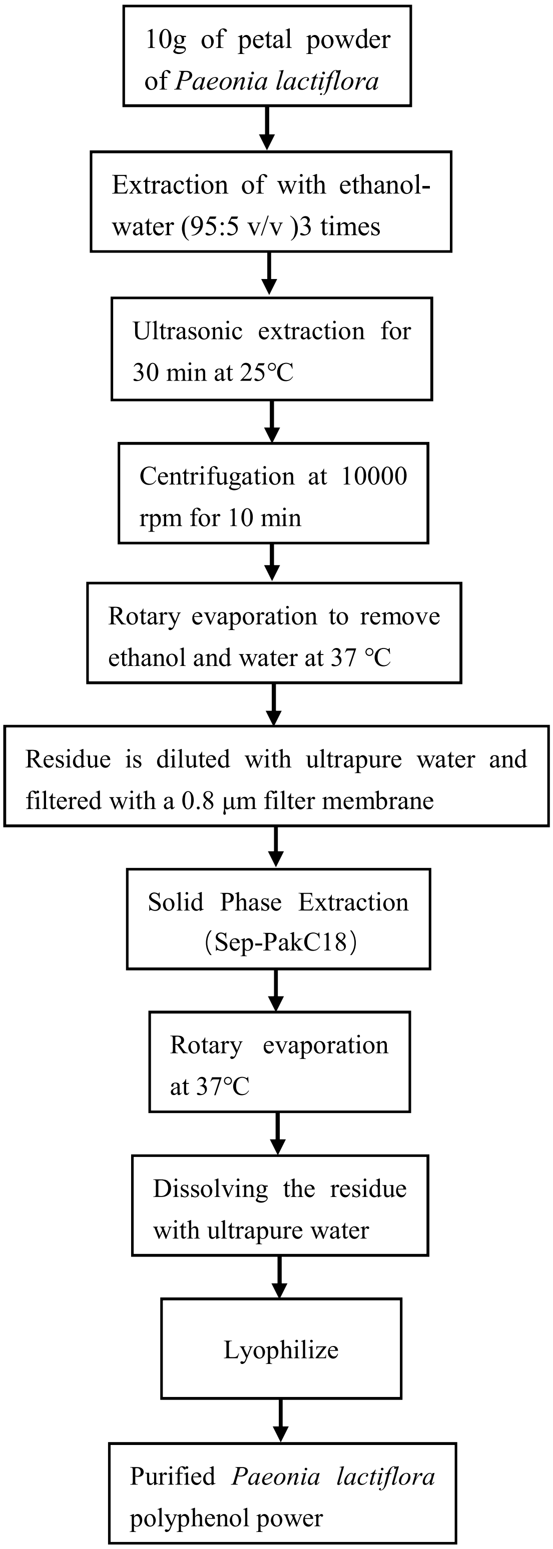

Figure S1. Diagram for the obtainment of purified *Paeonia lactiflora* petal flavonoids.

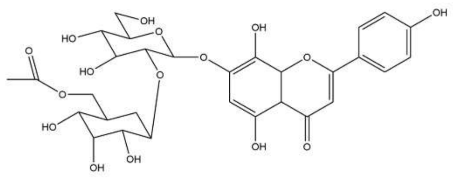

Isoscutellarein 7- (6'-acetylallosyl- (1->2) -glucoside) (**1**)

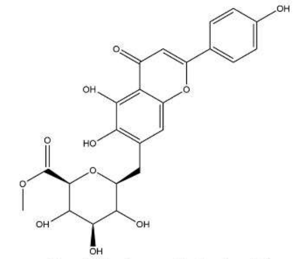

Scutellarin methylester (**2**)

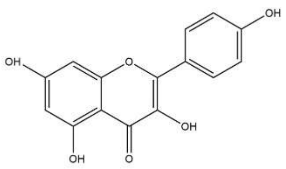

Kaempferol (**3**)

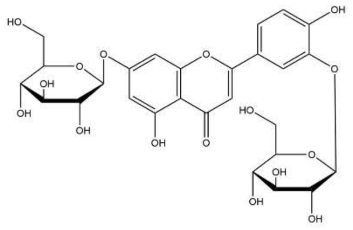

Luteolin-3',7-Diglucoside (**4**)

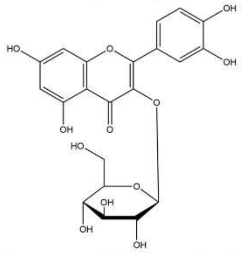

Quercetin-3*β*-D-glucoside (**5**)

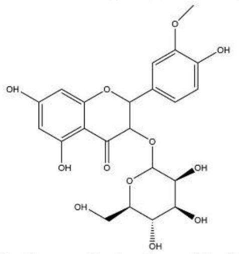

Isorhamnetin 3-galactoside (**6**)

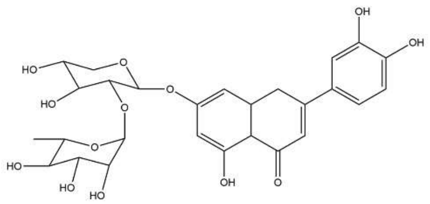

Luteolin 7- (6-malonylneohesperidoside) (**7**)

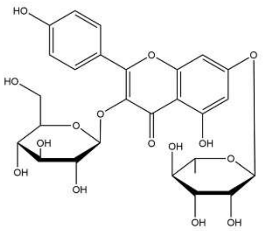

Kaempferol-3-*O*-*β*-glucopyranosyl-7-*O*-*α*-rhamnopyranoside (**8**)

Figure S2. Chemical structures of some main compounds in Paeonia lactiflora petal flavonoids

Table S1. Flavonoid compounds components in *Paeonia lactiflora* petal extract (PPF)

No.	Compounds	CAS	Molecular Formula	Concentration, μg/g extract
1	6-Methoxyluteolin	520-11-6	$C_{16}H_{12}O_7$	17.98
2	Diosmetin	520-34-3	C16H12O6	35.48
3	Quercetin	117-39-5	C15H10 O7	102.57
4	Naringenin	480-41-1	C15H12O5	12.60
5	Isorhamnetin	480-19-3	C16H12O7	78.46
6	Luteolin-3',7-Diglucoside	52187-80-1	C27H30O16	202.61
7	Kaempferol-3-O-glucorhamnoside	482-39-3	C21H20O10	10.28
8	Quercetin-3β-D-glucoside	482-35-9	C21H20O12	198.45
9	Kaempferol-3-O-β-glucopyranosyl-7-O-α-rhamnopyranoside	NA	C27H30O15	117.69
10	Kaempferol	520-18-3	C15H10O6	304.56
11	Cyanidin 3-O-glucoside	7084-24-4	C21H20O11	74.28
12	Quercetin 3-O-malonylglucoside	96862-01-0	C24H22O15	23.30
13	6-Methoxyluteolin 7-glucuronide methyl ester	NA	C23H22O13	82.50
14	Isoscutellarein 7- (6'-acetylallosyl- (1->2) -glucoside)	NA	C29H32O17	648.70
15	Isorhamnetin 3-galactoside	6743-92-6	C22H22O12	158.11
16	Dihydrokaempferol	480-20-6	C15H12O6	58.03
17	Scutellarin methylester	119262-68-9	C22H20O12	405.20
18	Luteolin 7- (6-malonylneohesperidoside)	NA	C30H32O18	121.23
19	Genistein	446-72-0	C15H10O5	4.44
20	Rhamnetin	90-19-7	C16H12O7	9.19
21	Okanin-4'-O-glucoside	535-96-6	C21H22O11	1.85
